# Bibliometric analysis of pancreatic cancer liver metastasis research: global trends, collaborations, and emerging research hotspots

**DOI:** 10.3389/fonc.2025.1546400

**Published:** 2025-07-15

**Authors:** Yingjie Huang, Zhijie Yin, Wei Han

**Affiliations:** Department of Pancreatic Surgery, The First Affiliated Hospital of Xinjiang Medical University, Urumqi, China

**Keywords:** pancreatic cancer, liver metastasis, bibliometric analysis (BA), global progress, research trends

## Abstract

**Background:**

Pancreatic cancer liver metastasis (PCLM) is a critical condition characterized by the spread of pancreatic cancer to the liver, significantly contributing to cancer-related mortality. The importance of understanding the epidemiology and research trends in PCLM cannot be overstated, as it impacts the development of effective treatment strategies and patient care in oncology.

**Method:**

This study provides a comprehensive epidemiological and bibliometric analysis of PCLM research. Advanced visualization tools such as VOSviewer, CiteSpace, and the R package “bibliometrix” were utilized to analyze the literature. A total of 3,941 publications were identified, covering a 15-year period from 2010 to 2024. The methodology included the identification of publication trends, country and institutional contributions, leading journals, and keyword co-occurrence analysis to uncover research hotspots.

**Results:**

The annual publication counts exhibited an upward trend, peaking at 384 in 2024, indicating a growing interest in PCLM research. The research included contributions from 90 countries and 4386 institutions, with China and the United States being the most prolific. The journal *Cancers* was identified as the most frequent publisher in this field. Keyword co-occurrence analysis revealed “cancer” and “pancreatic cancer” as key research hotspots, with a focus on prognostic factors and therapeutic strategies. The study also highlighted the importance of international collaboration and identified key contributors in the field.

**Conclusion:**

The findings of this study reveal the significant increase in PCLM research output, emphasizing the need for continued investigation and collaboration to improve treatment outcomes and patient care. While the study is limited by its reliance on bibliometric data without experimental validation, it nonetheless provides valuable insights into publication trends and emerging research themes in PCLM. The results underscore the importance of further research to enhance our understanding of this challenging area of oncology and to guide future research directions.

## Introduction

Pancreatic cancer (PC) is recognized as one of the most aggressive malignancies, characterized by a high mortality rate and poor prognosis (1). The epidemiological features of PC indicate a rising incidence and mortality rate globally, with projections suggesting a significant public health burden in the coming decades. Specifically, the incidence of PC is expected to reach 18.6 per 100,000 individuals by 2050, reflecting an annual growth of 1.1% (2, 3). This increase is attributed to various risk factors, including genetic predispositions, lifestyle choices, and environmental influences, which necessitate a comprehensive understanding of the disease for effective prevention and management strategies (4).

Liver metastasis is a common complication in patients with PC, occurring in approximately 39.96% of cases at the time of diagnosis (5). The presence of liver metastases significantly impacts the prognosis of PC patients, contributing to the disease’s lethality. The 5-year survival rate for metastatic PC is a mere 3%, underscoring the urgent need for advancements in early detection and therapeutic interventions (6). The mechanisms driving liver metastasis are multifaceted, involving interactions between cancer cells and the surrounding microenvironment, including immune cells, stromal components, and extracellular matrix elements. Systemic inflammation plays a key role not only in the development and progression of PC, but also in the formation of liver metastases (7). The inflammatory response not only promotes recruitment of immune cells, but also drives hepatic fibrosis and extracellular matrix (ECM) remodeling, creating a supportive structural environment for metastatic tumor cells (8). Immune dysregulation including immune evasion, formation of an immunosuppressive tumor microenvironment, and altered immune cell function create a favorable ecological niche for PC tumor growth and metastasis (9). In addition, in the case of PC (especially liver metastases), the sensitivity and specificity of early circulating tumor DNA (ctDNA) detection is critical for improving diagnostic accuracy and patient prognosis (10). Understanding the mechanisms underlying liver metastasis in PC is crucial for developing targeted therapies aimed at improving patient outcomes.

In this study, we employed bibliometric methods to systematically analyze the literature on PCLM, as well as to identify the current dominant research directions over the past 15 years (11). Our primary objective was to offer a comprehensive overview of research pertaining to PCLM. This involved examining trends in annual publication outputs, leading institutions and countries, the most productive authors, funding agencies, and the most frequently cited papers in the field. We aimed to provide a robust foundation for future in-depth research in this area.

## Methods

### Database and search strategy

**Figure 1:**
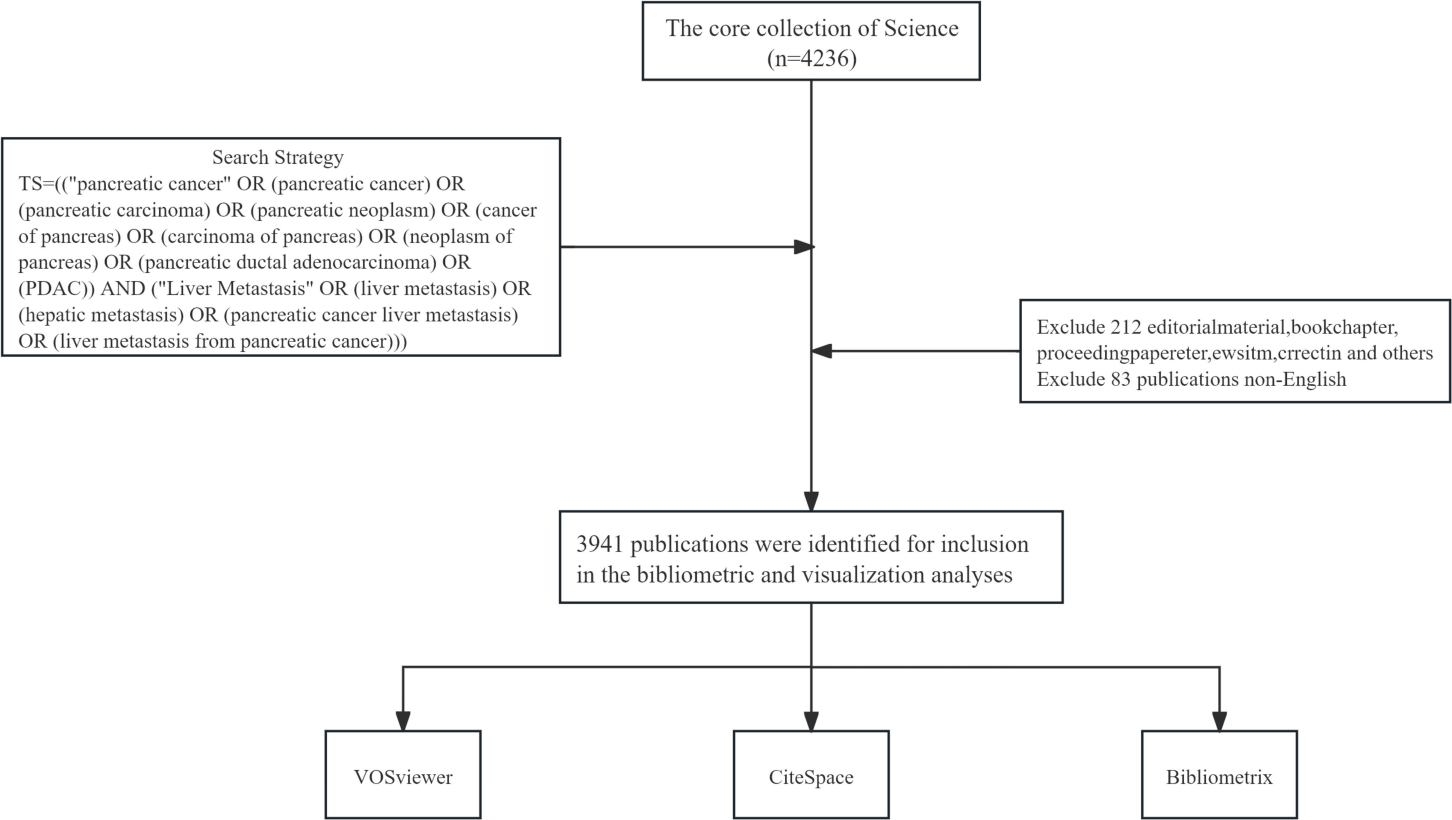
Publications screening flowchart.

We sourced the necessary publications for our study from the Web of Science Core Collection, an esteemed database encompassing over 21,100 peer-reviewed, high-quality academic journals from around the globe, including open-access titles. This comprehensive collection spans across more than 250 disciplines within the natural sciences, social sciences, arts, and humanities, and also incorporates conference proceedings and book chapters. In our study, we have selected English-language articles and reviews published between 1 January 2010, and 31 December 2024. The methodology of our search and the specific criteria for the inclusion and exclusion of studies are presented in **Figure 1**.

### Data analysis

VOSviewer (version 1.6.20) is a bibliometric software package that also functions as a tool for visualizing knowledge mapping. Utilizing fundamental classification clustering techniques, it enables the aggregation of literature keywords into clusters, thereby identifying research hotspots. In the knowledge map, “institution”, “journal”, and “author” are represented as nodes. The size of each node corresponds to the quantity of associated items, while the color denotes their respective classifications. The thickness of the lines connecting these nodes indicates the strength of collaboration or co-citation among them.

The R package “bibliometrix” (version 4.3.3) serves as a comprehensive tool for conducting quantitative research in the fields of scientometrics and bibliometrics (12). By employing this software, we executed a thematic evolution analysis to chart the development of research themes and concurrently developed a global network map of publications focused on PCLM.

CiteSpace is a software tool designed for the visualization and analysis of scientific literature. It enables researchers to dissect citation networks, author collaboration networks, and thematic evolution, thereby gaining a deeper understanding of the trends and focal points within their research domains. This study employed CiteSpace for the generation of overlay maps of journal bibliometrics, while Citation Bursts were applied to analyze the cited references, providing insights into the dynamics and focal points within the research domain.

## Results

### Quantitative analysis of publication

**Figure 2:**
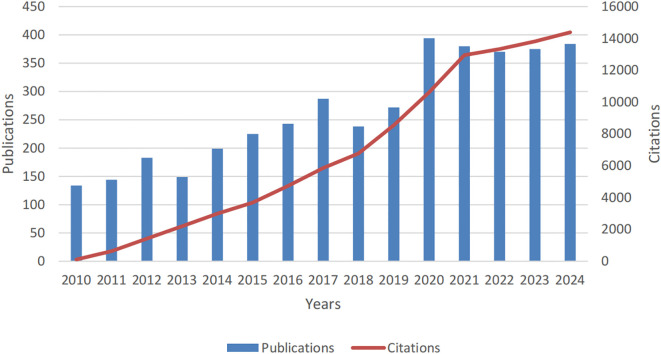
Annual publication volume and citation trends for studies on pancreatic cancer with liver metastases from 2010 to 2024.

Adhering to our defined search protocol, the literature review spanned a 15-year period and yielded 3,941 publications on PCLM, with 3,234 classified as original articles and 707 as review articles. As depicted in **Figure 2**, the annual publication count exhibited a significant upward trajectory from 2010 to 2024, escalating from 134 to 384. During this interval, the data revealed considerable fluctuations, with publication peaks of 394 in 2020 and a peak of 380 in 2021, contrasting with the lows of 134 in 2010 and 144 in 2011.

### Country and institutional analysis

**Figure 3:**
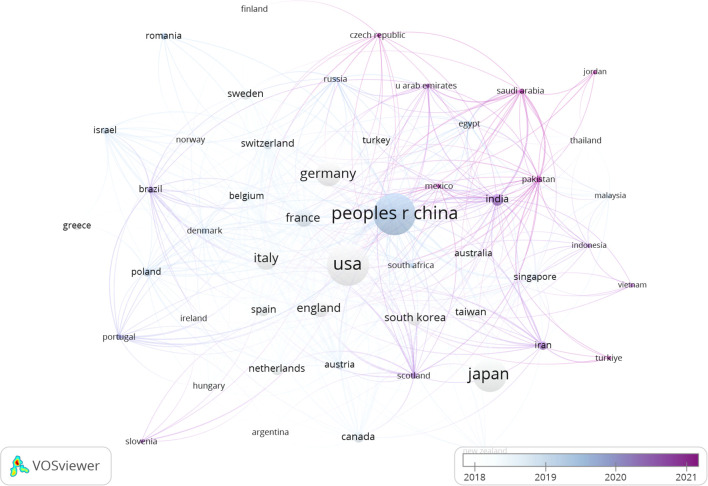
The visualization of countries on research of PCLM.

The publications spanned 90 countries and 4386 institutions. Within this global context, the United States stood out as the most prolific publisher with articles (n=1009), followed closely by China (n=1002), Japan (n=643), and Germany (n=380). The aggregate number of publications from China and the United States nearly constituted half of the overall total (**Table 1**). Subsequently, we filtered and visualized a selection of 50 countries with publication counts greater than or equal to 5, and constructed a collaboration network based on the number of publications and relationships among these countries. Notably, there was a significant amount of active collaboration between different countries (**Figure 3**).

**Table 1:** Top 10 countries and institutions on research of PCLM.

Rank	Country	Counts	Institution	Counts
1	The United States	1009	Fudan University	122
2	China	1002	Shanghai Jiao Tong University	79
3	Japan	643	Memorial Sloan Kettering Cancer Center	73
4	Germany	380	University of Texas MD Anderson Cancer Center	68
5	Italy	270	Zhejiang University	60
6	France	181	Johns Hopkins University	59
7	England	159	German Cancer Research Center	57
8	South Korea	143	Mayo Clinic	54
9	Spain	93	Sichuan University	51
10	Netherlands	87	University of California San Diego	49

**Figure 4:**
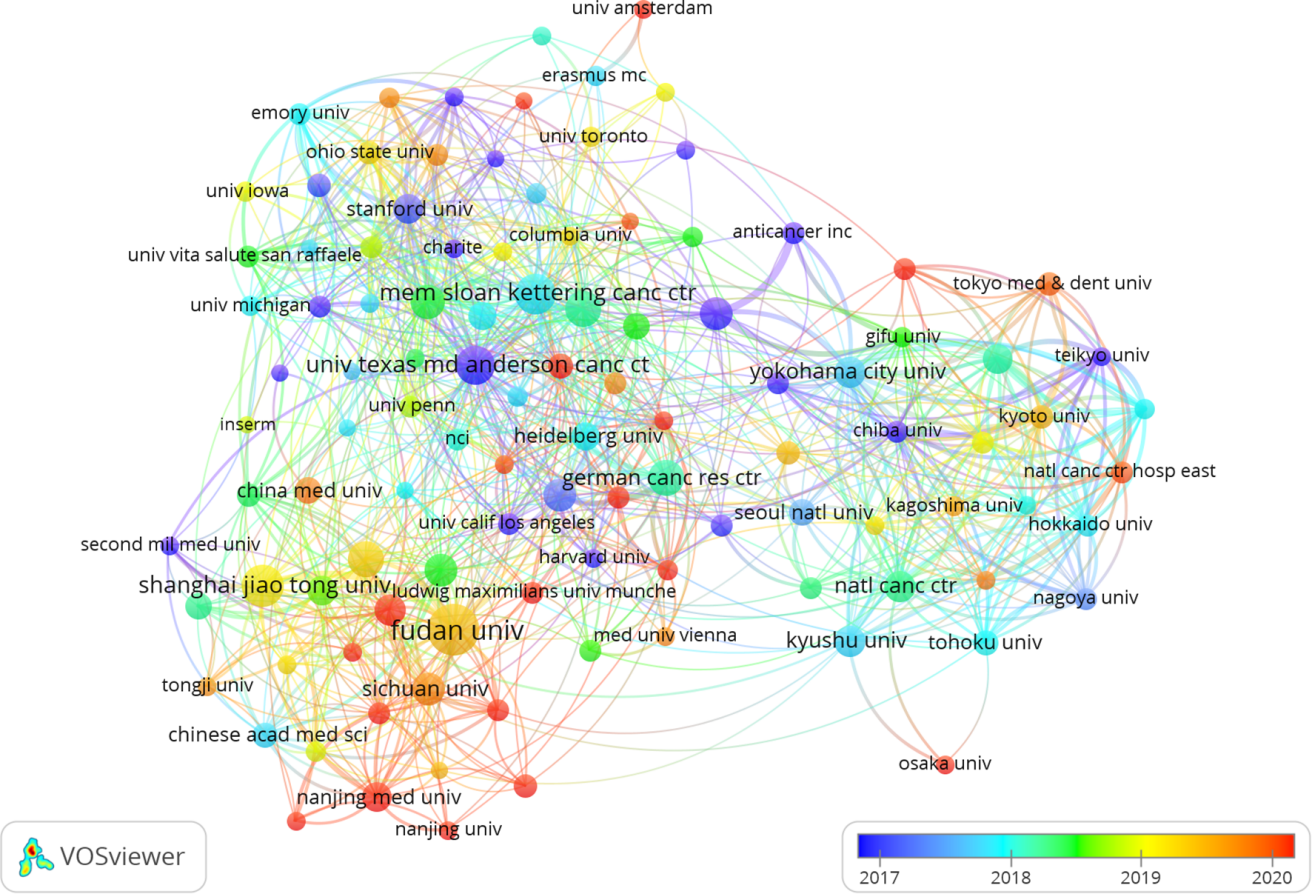
The visualization of institutions on research of PCLM.

Two out of the top ten ranked institutions are located in China. The top three institutions in terms of publication output are: Fudan University (n=122), Shanghai Jiao Tong University (n=79), and the Memorial Sloan Kettering Cancer Center (n=73). Subsequently, we selected 108 institutions based on a minimum publication threshold of 15 for visualization and constructed a collaboration network. Within this network, we observed particularly close collaborations between Fudan University, Shanghai Jiao Tong University, Johns Hopkins University, and University of Texas MD Anderson Cancer Center (**Figure 4**).

### Analysis of journals and co-cited journals

Publications pertaining to PCLM have been dispersed across 959 distinct academic journals. The journal with the highest number of publications on PCLM was *Cancers* (n=80), followed by *Annals of Surgical Oncology* (n=78), *Frontiers in Oncology* (n=67), *PLOS ONE* (n=55), and *Pancreatology* (n=54). Among the top 20 journals, the Journal of *Oncogene* had the highest impact factor (IF=6.9). Subsequently, we filtered 90 journals based on a minimum number of relevant publications equal to 10 and constructed a journal network graph (**Supplementary Figure 1A**). It revealed active citation relationships between *Cancers* and several other journals, including *PLOS ONE*, *Pancreatology*, *Pancreas*, and *Annals of Surgical Oncology*.

We further analyzed the co-cited journals and found that 6 journals had been cited more than 2500 times (**Table 2**). Among them, *Cancer Research* was the most frequently cited journal (Co-citation=4099), followed by *Journal of Clinical Oncology* (Co-citation=4042). Furthermore, we filtered journals with a minimum co-citation threshold of 100 to map the co-citation network, revealing active co-citation relationships between *Cancer Research* and *Journal of Clinical Oncology*, *Nature*, and *Annals of Surgery* (**Supplementary Figure 1B**).

**Table 2:** Top 10 journals and co-cited journals for research of PCLM.

Rank	Journal	Count	Co-cited journal	Co-citation
1	Cancers	80	Cancer Research	4099
2	Annals of Surgical Oncology	78	Journal of Clinical Oncology	4042
3	Frontiers in Oncology	67	Annals of Surgery	3284
4	PLOS ONE	55	Annals of Surgical Oncology	2789
5	Pancreatology	54	New England Journal of Medicine	2615
6	Pancreas	52	Clinical Cancer Research	2506
7	Medicine	50	Surgery	2114
8	Oncotarget	49	Pancreas	2102
9	World Journal of Gastroenterology	47	Nature	2012
10	Anticancer Research	45	PLOS ONE	1913

### Analysis of authors and co-cited authors

**Figure 5:**
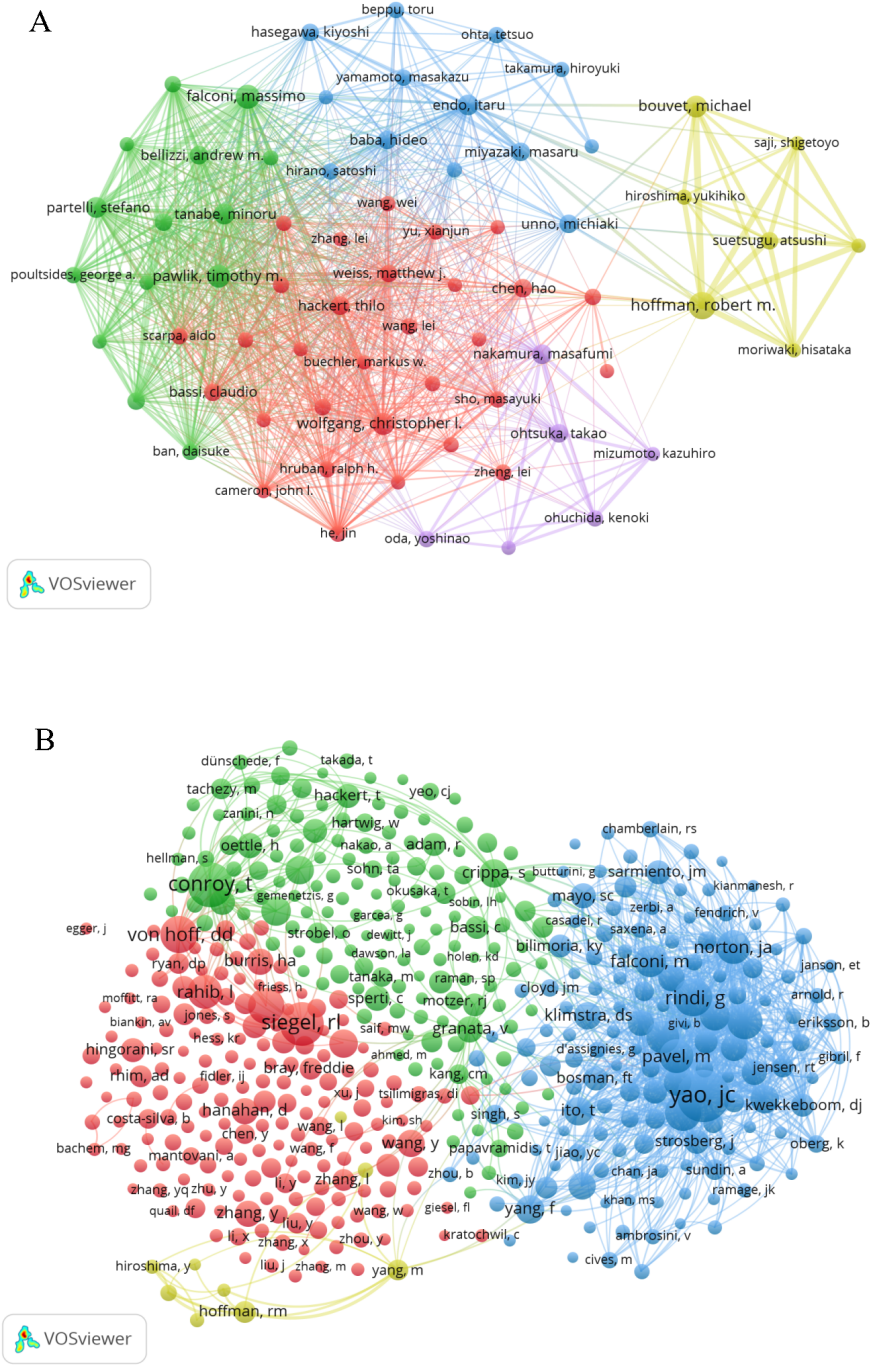
The visualization of authors **(A)** and co-cited Authors **(B)** on PCLM.

A total of 24,393 authors have contributed to the research on PCLM. Among the top 10 authors, each has published more than 15 papers (**Table 3**). We constructed a collaboration network (**Figure 5A**) based on authors with a publication count of at least 10. Notable nodes in this network include Hoffman RM, Wolfgang CL, Pawlik TM, and Bouvet M. Additionally, we observed close collaborations among several authors; for instance, Endo I, Nakamura M, and Falconi M have actively collaborated.

**Table 3:** Top 10 authors and co-cited authors on research of PCML.

Rank	Authors	Count	Co-cited authors	Citations
1	Hoffman RM et al	34	Yao JC et al	587
2	Falconi M et al	26	Conroy T et al	439
3	Pawlik TM et al	24	Siegel RL et al	420
4	Wolfgang CL et al	22	Rindi G et al	315
5	Bouvet M et al	22	Von Hoff DD et al	300
6	Endo I et al	20	Modlin IM et al	288
7	Partelli S et al	19	Jemal A et al	288
8	Tanabe M et al	19	Kulke MH et al	274
9	Nakamura M et al	17	Pavel M et al	251
10	Ohtsuka T et al	16	Norton JA et al	246

Among the 73,450 co-cited authors, four authors had more than 300 citations each (**Table 3**). The most frequently co-cited author was Yao JC (n=587), followed by Conroy T (n=439) and Siegel RL (n=420). By filtering authors with a minimum of 30 co-citations, we constructed a co-citation network graph (**Figure 5B**). Within this network, we observed active collaborations among different co-cited authors, such as Conroy T, Siegel RL, and Von Hoff DD.

### Analysis of co-cited references

**Figure 6:**
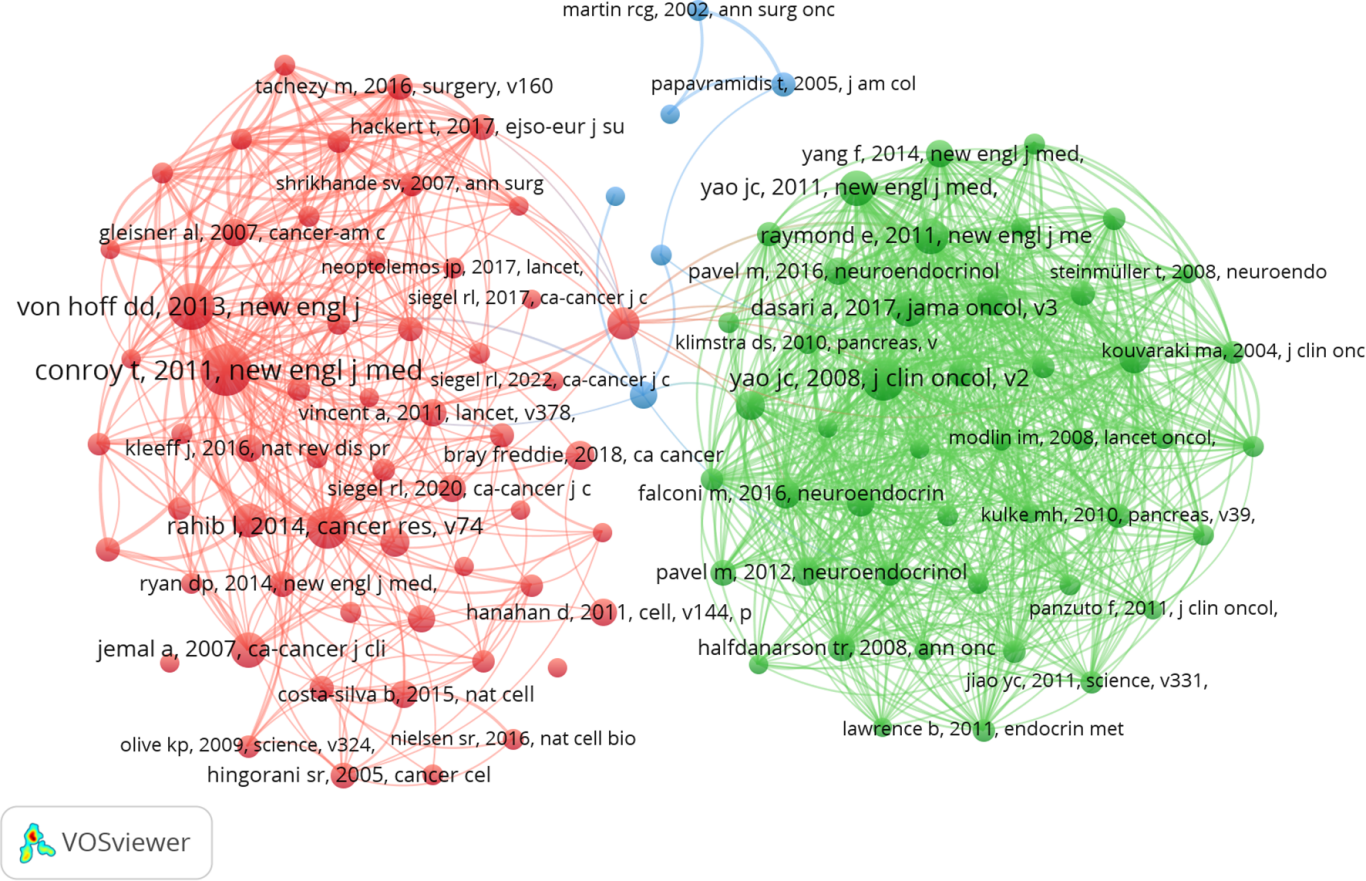
The visualization of co-cited references on research of PCLM.

A total of 119,617 co-cited documents pertain to research on PCLM. Among the top 10 co-cited references, each reference was cited at least 120 times (**Table 4**). We selected references with a co-citation count of at least 40 to construct a co-citation network graph (**Figure 6**). This graph illustrates active co-citation relationships among “Conroy T, 2011, New England Journal of Medicine”, “Rahib I, 2014, Cancer Res”, and “Von Hoff DD, 2013, New England Journal of Medicine”.

**Table 4:** Top 10 co-cited references on research of PCLM.

Rank	Co-cited reference	Citations
1	Conroy T, 2011, New England Journal of Medicine, V364, P1817	333
2	Von Hoff DD, 2013, New England Journal of Medicine, V369, P1691	275
3	Yao JC, 2008, Journal of Clinical Oncology, V26, P3063	229
4	Rahib I, 2014, Cancer Research, V74, P2913	228
5	Dasari A, 2017, JAMA Oncology, V3, P1335	154
6	Raymond E, 2011, New England Journal of Medicine, V364, P501	152
7	Yao JC, 2011, New England Journal of Medicine, V364, P514	150
8	Jemal A, 2007, CA-A CANCER JOURNAL FOR CLINICIANS, V57, P43	148
9	Burris HA, 1997, Journal of Clinical Oncology, V15, P2403	143
10	Eisenhauer EA, 2009, European Journal of Cancer, V45, P228	121

### Analysis of reference with citation bursts

**Figure 7:**
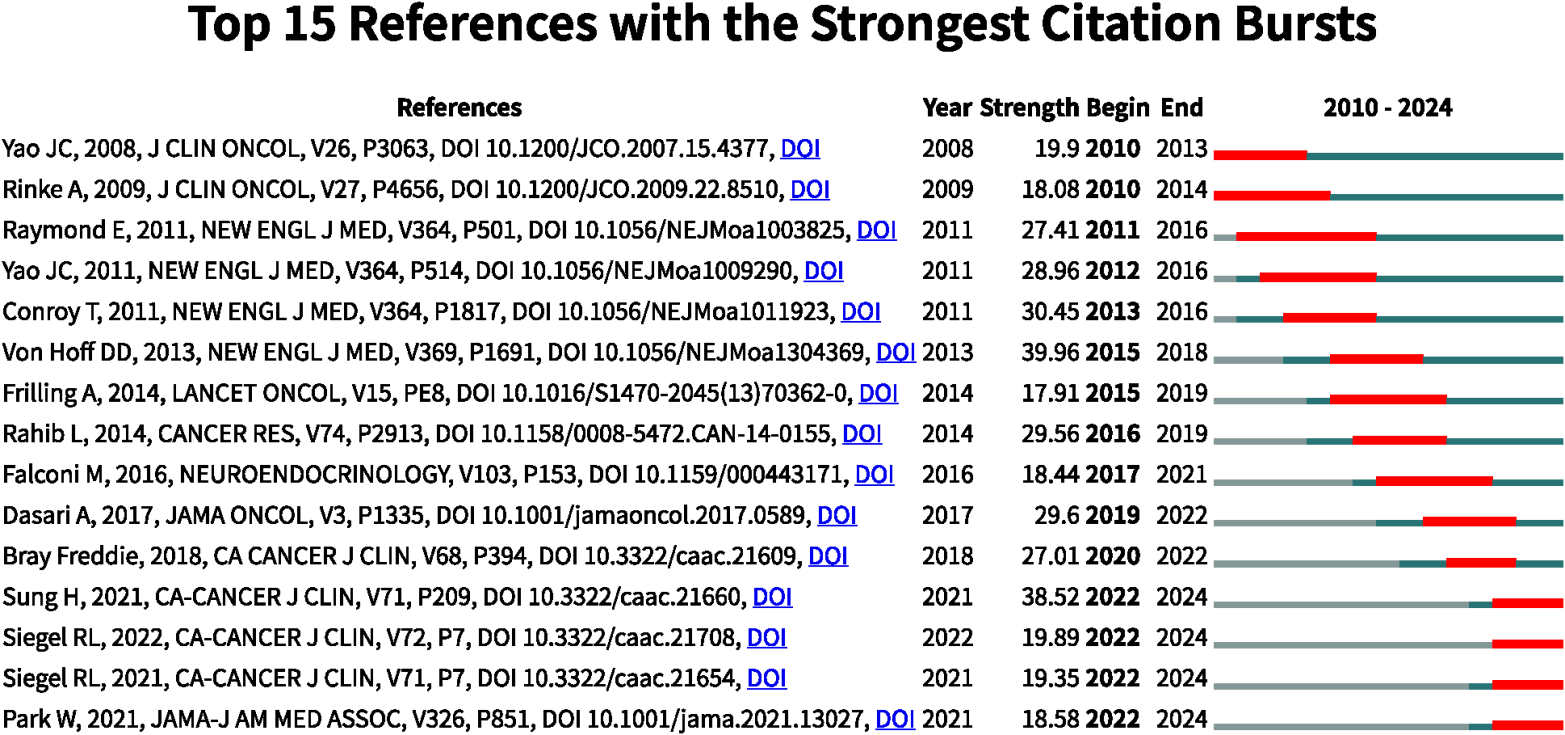
Top 15 references with strong citation bursts.

References exhibiting citation bursts are those that have been frequently cited by scholars within a specific field during a defined period. In our study, CiteSpace identified 15 references with significant citation bursts (**Figure 7**). As depicted in **Figure 7**, each bar represents a year, with red bars indicating strong citation bursts. Citation bursts first emerged in 2010 and the latest occurred in 2024. The reference with the highest burst strength (strength = 39.96) is “Von Hoff DD, 2013, N Engl J Med”. **Table 5** summarizes the main findings of the 15 references in the order they appear in **Figure 7**.

**Table 5:** The main research contents of the 15 references with strong citations bursts.

Rank	Strength	Reference
1	19.9	One hundred years after “carcinoid”: epidemiology of and prognostic factors for neuroendocrine tumors in 35,825 cases in the United States
2	18.08	Placebo-controlled, double-blind, prospective, randomized study on the effect of octreotide LAR in the control of tumor growth in patients with metastatic neuroendocrine midgut tumors: a report from the PROMID Study Group
3	27.41	Sunitinib malate for the treatment of pancreatic neuroendocrine tumors
4	28.96	Everolimus for advanced pancreatic neuroendocrine tumors
5	30.45	FOLFIRINOX versus gemcitabine for metastatic pancreatic cancer
6	39.96	Increased survival in pancreatic cancer with nab-paclitaxel plus gemcitabine
7	17.91	Recommendations for management of patients with neuroendocrine liver metastases
8	29.56	Projecting cancer incidence and deaths to 2030: the unexpected burden of thyroid, liver, and pancreas cancers in the United States
9	18.44	ENETS Consensus Guidelines Update for the Management of Patients with Functional Pancreatic Neuroendocrine Tumors and Non-Functional Pancreatic Neuroendocrine Tumors
10	29.6	Trends in the Incidence, Prevalence, and Survival Outcomes in Patients With Neuroendocrine Tumors in the United States
11	27.01	ENETS Consensus Guidelines Update for the Management of Patients with Functional Global cancer statistics 2018: GLOBOCAN estimates of incidence and mortality worldwide for 36 cancers in 185 countries.
12	38.52	Global Cancer Statistics 2020: GLOBOCAN Estimates of Incidence and Mortality Worldwide for 36 Cancers in 185 Countries
13	19.89	Cancer statistics, 2022
14	19.35	Cancer statistics, 2021
15	18.58	Pancreatic Cancer: A Review

### Analysis of hotspots and frontiers

**Figure 8:**
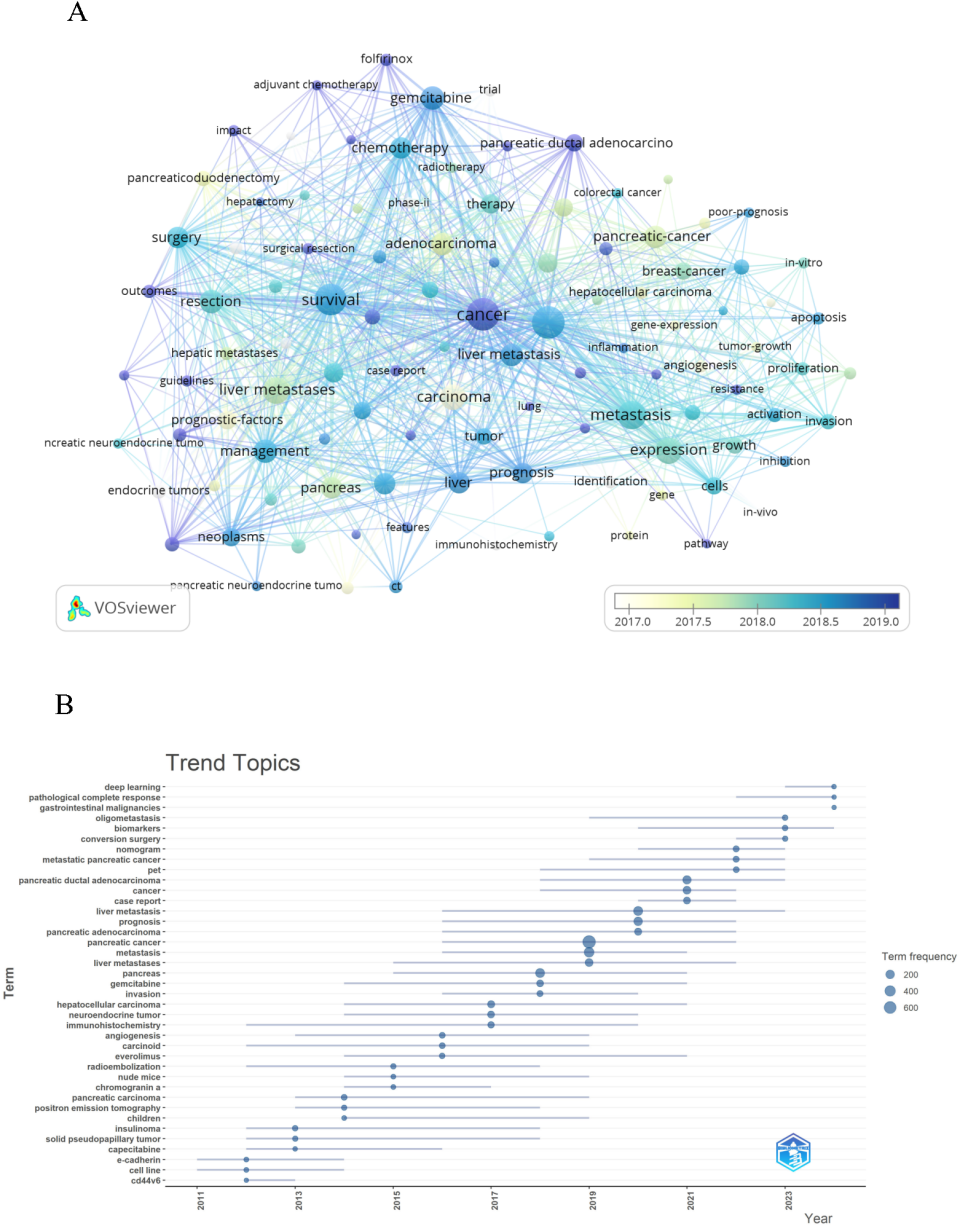
Keyword cluster analysis **(A)** and trend topic analysis **(B)**.

By conducting co-occurrence analysis of keywords, we rapidly identified research hotspots in the field of PCLM. **Table 6** displays the top 20 high-frequency keywords in PCLM research. We filtered keywords with occurrence counts greater than or equal to 50 and conducted cluster analysis using VOSviewer (**Figure 8A**). The thicker the lines between nodes, the stronger the association between the keywords. The trend analysis of keywords from 2010 to 2024 is depicted in **Figure 8B**, indicating that current research is primarily focused on areas such as deep learning and conversion surgery.

**Table 6:** Top 20 keywords on research of PCLM.

Rank	Keywords	Counts	Rank	Keywords	Counts
1	Cancer	739	11	Liver metastasis	329
2	Pancreatic cancer	724	12	Adenocarcinoma	320
3	Survival	689	13	Pancreatic-cancer	316
4	Metastases	566	14	Liver	307
5	Expression	478	15	Chemotherapy	304
6	Liver metastases	460	16	Pancreas	300
7	Carcinoma	424	17	Diagnosis	299
8	Gemcitabine	376	18	Surgery	288
9	Management	360	19	Prognosis	276
10	Resection	359	20	Hepatocellular-carcinoma	255

## Discussion

### Global research status and trends

This study provides a comprehensive bibliometric analysis of research trends related to PCLM, highlighting the growing academic interest over the past 15 years. By employing advanced visualization tools such as VOSviewer and CiteSpace, we identified key contributors, publication patterns, and emerging research hotspots in the field. This study provides an objective and systematic analysis of the current research status, development trends, and future research hotspots of PCLM. It aims to help scholars gain a quick understanding of the research landscape in this field and offers valuable directions for topic selection activities. The study begins by analyzing publication trends, including countries, institutions, authors, and journals. Subsequently, keywords are clustered and analyzed to identify the prevailing research hotspots in this specialized area.

An analysis of publication trends shows a significant increase in studies on liver metastases from pancreatic cancer after 2020, with such studies accounting for 48.5% of the total number of papers. This reflects researchers’ intense interest in comprehending and probing into this aggressive cancer. Nevertheless, research on PCLM is still in want of effective therapies and breakthroughs. Thus, it is imperative that more countries and researchers join forces in future investigations.

In terms of geographical contributions, PCLM research spans 90 countries, with China and the United States being the most prolific contributors. The institution with the highest number of publications is Fudan University in China. The number of citations is a common indicator of professional recognition of scientific work, so citation analysis is widely used to assess the quality of research work. The fact that the most cited papers are in the United States suggests that the United States is a major player and a world leader in this field; despite the large number of papers published in China, the citation rate is very low, suggesting that China must improve the quality and impact of its research. In addition, both China and the United States are major cancer research countries, and international collaboration has played a key role in this regard. These joint efforts have not only facilitated the sharing of knowledge and resources, but also fostered innovation, which has been crucial in addressing the multifaceted challenges posed by PCLM.

In the topic trend graph (**Figure 8B**), we found that the keywords “deep learning”, “biomarkers”, “conversion surgery” and “gemcitabine Bin” are more significant. It indicates that the study of deep learning and the exploration of surgical treatments have been a hotspot of research. In addition, chemotherapy-related studies have gradually occupied an important position in recent years, and these studies are often closely integrated with prognostic analysis. We explore the research hotspots and directions in the field of PCLM by analyzing the topic trends (**Table 7**).

**Table 7:** The representative research related to thematic trend terms.

Rank	Keywords	Researches
1	Deep learning	A novel model for predicting postoperative liver metastasis in R0 resected pancreatic neuroendocrine tumors: integrating computational pathology and deep learning-radiomics (13)
		Identification of Pancreatic Metastasis Cells and Cell Spheroids by the Organelle-Targeting Sensor Array (14)
		Prometheus: A Risk Classification Deep Learning Model for Predicting Liver Metastases from Pancreatic Cancer (15)
2	Biomarkers	Quantitative Imaging Biomarkers of the Whole Liver Tumor Burden Improve Survival Prediction in Metastatic Pancreatic Cancer (16)
		Evaluation of circulating tumor DNA as a biomarker in pancreatic cancer with liver metastasis (17)
		Biomarkers in Liquid Biopsies for Prediction of Early Liver Metastases in Pancreatic Cancer (18)
3	Chemotherapy & Conversion Surgery	Metastatic pancreatic cancer with complete response to FOLFIRINOX treatment (19)
		The prognostic impact of tumour location and first-line chemotherapy regimen in locally advanced pancreatic cancer (20)
		Study protocol of an open-label, single arm phase II trial investigating the efficacy, safety and quality of life of neoadjuvant chemotherapy with liposomal irinotecan combined with Oxaliplatin and 5-fluorouracil/Folinic acid followed by curative surgical resection in patients with hepatic Oligometastatic adenocarcinoma of the pancreas (HOLIPANC) (21)
		A systematic review of surgical resection of liver-only synchronous metastases from pancreatic cancer in the era of multiagent chemotherapy (22)
		Simultaneous resection of the primary tumour and liver metastases after conversion chemotherapy versus standard therapy in pancreatic cancer with liver oligometastasis: protocol of a multicenter, prospective, randomized phase III control trial (CSPAC-1) (23)
		Surgical management of pancreatic cancer liver oligometastases (24)
		Treatment of oligo-metastatic pancreatic ductal adenocarcinoma to the liver: is there a role for surgery? A narrative review (25)

### Deep learning

Deep learning, a subset of machine learning, has gained significant traction in the medical field, particularly due to its ability to analyze complex data patterns and automate processes that were previously reliant on human expertise. At the heart of deep learning are neural networks, which are computational models inspired by the architecture of the human brain. The most common types of neural networks used in medical applications are Convolutional Neural Networks (CNNs) and Recurrent Neural Networks (RNNs). According to the study, a new predictive model integrating computational pathology scoring and deep learning-radiomics can better predict postoperative liver metastases in patients with pan-NET, thus helping clinicians develop personalized treatment plans (13). The prediction of PCLM has gained significant attention due to its critical implications for patient management and treatment outcomes. The data sources for these predictive models are multifaceted, encompassing clinical, imaging, and genetic information. Clinical data are usually derived from large retrospective cohorts, and the use of databases allows for the identification of key clinical risk factors associated with liver metastases, such as tumor size, histological subtype, and the presence of other metastases, which are essential for the development of predictive nomograms (26, 27). In addition, genetic and molecular data have become valuable components in the field of risk prediction. The development of a liver metastasis score (LMS) based on differentially expressed genes has been proposed as a new approach to stratify patients according to their risk of developing liver metastases (28).

### Biomarkers

Research into the biological mechanisms underlying pancreatic cancer metastasis has revealed that factors such as the tumor microenvironment, molecular signaling pathways, metabolic alterations, and immune evasion play pivotal roles in the metastatic process. The tumor microenvironment, characterized by a complex interplay between cancer cells and surrounding stromal and immune cells, significantly influences tumor behavior and progression. Furthermore, dysregulation of key molecular pathways, including those involved in cell proliferation, invasion, and metastasis, has been identified as a critical factor in the transition from localized disease to distant metastasis. Understanding these mechanisms is essential for the development of targeted therapies and the identification of novel biomarkers that can aid in the early detection of liver metastasis.

Recent advances in liquid biopsy techniques, particularly the analysis of circulating tumor DNA (ctDNA), have shown promise in monitoring disease progression and treatment response in PC (29). The ability to capture ctDNA reflects the tumor’s genetic landscape, allowing for a more comprehensive understanding of the disease state and facilitating personalized treatment approaches (30). With the development of cancer diagnosis, ctDNA has become a key biomarker for the early detection of liver metastasis, recurrence monitoring and prognostic assessment of cancer (31).

The immune microenvironment plays a pivotal role in the progression and metastasis of PC, particularly in liver metastasis, which is a common and detrimental outcome in this malignancy. PC is characterized by a unique immune landscape that is often immunosuppressive, contributing to its aggressive nature and poor prognosis. The tumor microenvironment is heavily infiltrated by various immune cells, including regulatory T cells (Tregs), myeloid-derived suppressor cells (MDSCs), and tumor-associated macrophages (TAMs), which collectively create a barrier to effective anti-tumor immunity. This immunosuppressive environment is further exacerbated by the presence of inflammatory cytokines and chemokines that promote tumor growth and metastasis. the application of Mendelian randomization analysis has enabled researchers to explore the causal relationship between genetic variants associated with immune responses and the risk of inflammation and cancer (32, 33).

The gut microbiome has emerged as a critical player in the pathogenesis and progression of various cancers, including PCLM. Ecological dysregulation or imbalance of the gut microbiota has been linked to the pathogenesis of PC, suggesting a complex interaction between microbial composition and tumor development (34). Recent studies have demonstrated that the composition of the gut microbiome can influence tumor behavior, immune responses, and treatment outcomes. Probiotics and microbiome-targeted therapies are gaining attention as potential adjunctive treatments for PCLM, as they may help modulate the tumor microenvironment and enhance the efficacy of existing therapies. For instance, a study found that certain gut bacteria, such as Streptococcus, were significantly enriched in PC patients and correlated with disease progression, suggesting that these microorganisms could serve as biomarkers for early detection and therapeutic targets (35). Furthermore, the modulation of the gut microbiome has been shown to influence the metabolism of therapeutic agents, potentially improving treatment outcomes for patients with PCLM (36).

### Chemotherapy and conversion surgery

Conversion surgery has emerged as a crucial strategy in the management of patients with initially unresectable PC, particularly in those with liver metastases. The evolution of chemotherapy regimens has had a significant impact on the management of PC. Current mainstream chemotherapy regimens, such as FOLFIRINOX and gemcitabine with nab-paclitaxel, have demonstrated significant improvements in tumor response rates and progression-free survival, thus creating opportunities for conversion surgery (37). The introduction of neoadjuvant chemotherapy has revolutionized the treatment landscape, allowing for better patient selection for surgical intervention. Studies have shown that patients who undergo conversion surgery after responding to chemotherapy can experience improved overall survival rates compared to those who receive only palliative care (38). While conversion surgery holds promise for select patients with PC, its limitations and challenges are substantial. The low conversion rates, coupled with the high risks of surgical intervention and the complexities of preoperative and intraoperative assessments, highlight the need for continued research and refinement of surgical techniques. Future studies should focus on identifying biomarkers that can better predict which patients are likely to benefit from conversion surgery, as well as developing innovative surgical approaches that minimize complications and maximize the chances of successful resection (39).

### Limitations

Limitations of this study mainly stem from the reliance on bibliometric data, the only database included in the literature was WOS, the citation-based analysis may have prioritized older or English-medium papers, and the lack of experimental or clinical validation in this study, which may affect the robustness of the findings. The nature of bibliometric analysis may introduce biases, as it is contingent upon the availability and accuracy of published literature. Furthermore, the interpretation of research trends may be confounded by varying publication practices across different countries and institutions, leading to discrepancies in the perceived impact of specific research contributions. Future work should combine bibliometrics with multi-omics or patient-level data to overcome these shortcomings. These factors underscore the necessity for complementary studies that integrate experimental and clinical insights to validate the bibliometric findings and enhance the comprehensive understanding of PCLM.

## Conclusion

In conclusion, the significant rise in research output pertaining to PCLM underscores the growing recognition of this critical area within oncology. Our findings highlight the importance of international collaboration and the identification of research hotspots that can inform future investigations and therapeutic strategies. By directing attention and resources toward these emerging areas, the oncology community can foster advancements that ultimately contribute to improved patient outcomes in this challenging disease context.

## Data Availability

Publicly available datasets were analyzed in this study. This data can be found here: https://www.webofscience.com/wos/.
