# Supplementary material for: Bibliometric analysis of pancreatic cancer liver metastasis research: global trends, collaborations, and emerging research hotspots
Source: Front Oncol. 2025 Jul 15;15:1546400. doi: 10.3389/fonc.2025.1546400 (PMC12303823; doi:10.3389/fonc.2025.1546400)
Supplement: Supplementary file 1 [file DataSheet1.pdf]

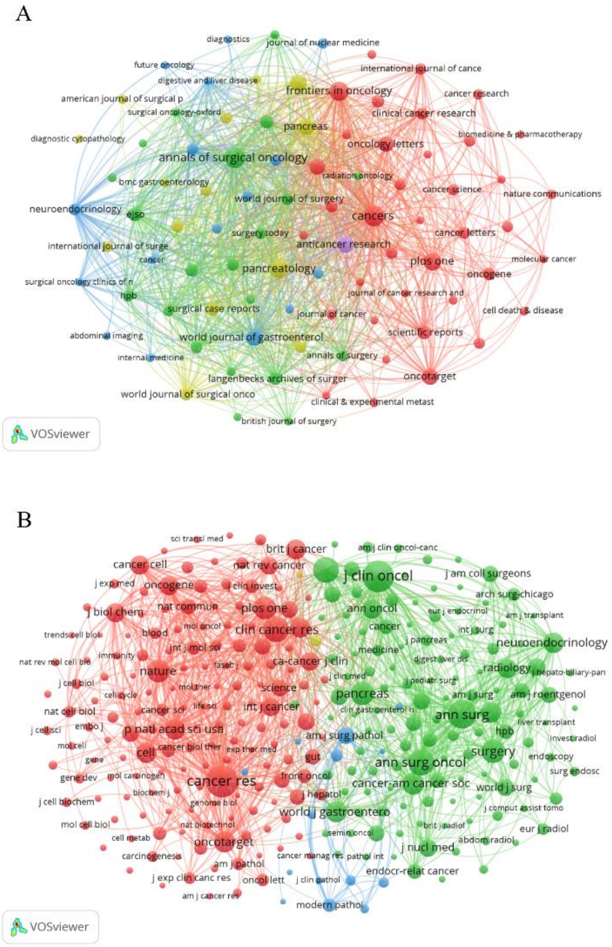

Supplementary Figure 1 The visualization of journals (A) and co-cited journals (B) on research of PCLM.
